# Synaptic membrane rafts: traffic lights for local neurotrophin signaling?

**DOI:** 10.3389/fnsyn.2013.00009

**Published:** 2013-10-18

**Authors:** Barbara Zonta, Liliana Minichiello

**Affiliations:** Department of Pharmacology, University of OxfordOxford, UK

**Keywords:** neurotrophin signaling, lipid rafts, synaptic rafts, BDNF, TrkB receptor translocation

## Abstract

Lipid rafts, cholesterol and lipid rich microdomains, are believed to play important roles as platforms for the partitioning of transmembrane and synaptic proteins involved in synaptic signaling, plasticity, and maintenance. There is increasing evidence of a physical interaction between post-synaptic densities and post-synaptic lipid rafts. Localization of proteins within lipid rafts is highly regulated, and therefore lipid rafts may function as traffic lights modulating and fine-tuning neuronal signaling. The tyrosine kinase neurotrophin receptors (Trk) and the low-affinity p75 neurotrophin receptor (p75^NTR^) are enriched in neuronal lipid rafts together with the intermediates of downstream signaling pathways, suggesting a possible role of rafts in neurotrophin signaling. Moreover, neurotrophins and their receptors are involved in the regulation of cholesterol metabolism. Cholesterol is an important component of lipid rafts and its depletion leads to gradual loss of synapses, underscoring the importance of lipid rafts for proper neuronal function. Here, we review and discuss the idea that translocation of neurotrophin receptors in synaptic rafts may account for the selectivity of their transduced signals.

## Introduction

Neurons are highly polarized cells. They are organized in distinct morphological regions, namely cell soma, dendrites and axon. Each of these regions performs specific functions. In particular, dendrites receive information from other neurons, whereas the axon conveys information from one neuron to another through connections known as synapses. The axon-dendrite polarity ensures unidirectional transmission of the signal from pre-synaptic cells to post-synaptic targets (Barnes and Polleux, 2009). The synapses are highly specialized subdomains. It is clear by now that heterogeneous multiprotein signaling complexes assemble at pre- and post-synaptic parent neurons thus providing diversity to the molecular composition and functional properties of chemical synapses in the mammalian central nervous system (CNS) (O'Rourke et al., 2012).

One key question is how proteins are specifically recruited to these compartments.

It is widely accepted that proteins and lipids do not freely diffuse along cell membranes as initially proposed (Singer and Nicolson, 1972); instead their lateral mobility is constrained. After observing heterogeneity in lipid phases of cell membranes, it has been suggested that lipids are organized into domains, also known as lipid rafts, which are enriched in sphingolipids and cholesterol (Karnovsky et al., 1982). The presence of cholesterol makes lipid domains more insoluble than the rest of the membrane and, after differential centrifugation in density gradients, they can be isolated from soluble fractions (Simons and Toomre, 2000). Lipid rafts have been proposed to function as platforms orchestrating the specific lipid-protein and protein-protein interactions that activate cellular processes, including signal transduction, cell-adhesion, membrane trafficking and molecular sorting. In particular, they are believed to localize signaling molecules, allowing these to interact with each other but not with those that are excluded from the rafts (Simons and Toomre, 2000; Allen et al., 2007; Lingwood and Simons, 2010; Simons and Sampaio, 2011).

However, the raft concept has undergone severe scrutiny. Since its formal introduction in 1982 (Karnovsky et al., 1982), the concept of membrane rafts has been quite controversial due to the difficulty in characterizing the cell membrane structure. Initially, the controversy stemmed from the employment of indirect methods to prove the existence of lipid rafts. These included detergent extraction protocols to isolate insoluble membrane fractions, which turned out to be highly variable in composition depending on the type of detergent, the time of extraction and the temperature. Additionally, cholesterol depletion experiments have implicated lipid rafts in processes where the results reflected more global perturbations of the cell membrane than specific effects (Munro, 2003; Simons and Gerl, 2010).

The advancement in imaging technology, lipidomic analysis and biophysical assays has allowed to directly visualize lipid rafts in living cells and to define their characteristics using primarily synthetic membranes (Simons and Gerl, 2010). More specifically, the concept of lipid rafts has evolved to include short-lived and highly dynamic structures that exist as nanoscale assemblies, in which components are either permanently or just transiently associated. Activation of distinct signaling pathways would depend on their protein/lipid composition and cell type (Pike, 2003; Lingwood and Simons, 2010).

Despite persisting skepticism among scientists the appealing nature of the lipid raft concept has prompted researchers to hypothesize possible biological functions of lipid rafts at synapses. This is based on the observation that detergent resistant synaptic membranes (i.e., synaptic rafts) are enriched in cholesterol and proteins, including neurotransmitter receptors, signaling molecules and cytoskeletal-adaptor proteins (Suzuki, 2002). In addition, there is increasing evidence of a physical interaction between post-synaptic densities and post-synaptic lipid rafts (Suzuki et al., 2011). The latter have also been proposed to play a role in membrane trafficking and receptor sorting near synaptic active sites (Torres et al., 1998; Bruckner et al., 1999; Suzuki et al., 2011). Moreover, the importance of synaptic membrane rafts is underscored by the fact that their disruption leads to depletion of synapses, loss of dendritic spines and instability of neurotransmitter receptors (Hering et al., 2003).

The neurotrophin Trk receptors and the p75^NTR^ low affinity receptor were found enriched in neuronal lipid rafts together with the intermediates of downstream signaling pathways, suggesting a possible role of lipid rafts in neurotrophin signaling (Higuchi et al., 2003; Suzuki et al., 2004). Various models have been proposed for the involvement of lipid rafts in neurotrophin signaling in the context of cell survival and neurite outgrowth (Paratcha and Ibanez, 2002; Suzuki et al., 2011). Here, we aim to review recent evidence supporting the idea that compartmentalization of neurotrophin receptors in lipid rafts at synapses may account for the selectivity of their transduced signals.

## Neurotrophin signaling at synaptic rafts

The neurotrophin family of growth factors, including brain derived neurotrophic factor (BDNF) and nerve growth factor (NGF), are diffusible molecules synthesized as precursors (pro-neurotrophins) and subsequently cleaved by proteases and convertases to produce mature neurotrophins. Pro-neurotrophins preferentially binds to the p75^NTR^ receptor, a single transmembrane protein of the superfamily of tumor necrosis factor (TNF) receptors, whose activation is usually associated with apoptosis (Lee et al., 2001; Lu et al., 2005).

In their mature form, BDNF and NGF bind with high affinity to TrkB and TrkA receptors, respectively, members of the tyrosine kinase family of receptors. The signaling pathways activated by these two receptors promote survival, differentiation, axon growth, dendrite pruning and other aspects of nervous system maturation and function (Chao, 2003).

BDNF has been the most extensively studied among the neurotrophins and found to play, among other biological functions, a pivotal role in synaptic transmission and activity-dependent synaptic plasticity (Minichiello, 2009; Edelmann et al., 2013). A very active research area is focused on the role of BDNF in modulating synaptic efficacy at specific synaptic sites (Poo, 2001). One fascinating idea is that partitioning of neurotrophin receptors in synaptic rafts may explain the selectivity of their transduced signals. Importantly, the receptors mediating BDNF signaling have been found to localize in lipid rafts together with the effectors of their downstream signaling pathways (Higuchi et al., 2003; Suzuki et al., 2004; Pereira and Chao, 2007).

## Lipid raft regulation of BDNF signaling at synapses

BDNF/TrkB signaling is known to activate multiple transduction pathways, which include the phosphatidylinositol 3–kinase (PI3K)–AKT, ERK/MAPK, and the phospholipase C-γ (PLC-γ) pathways. In principle the three pathways can work in parallel and have all been implicated in controlling various aspect of synaptic plasticity, including translation, transport and translocation of synaptic proteins (Yoshii and Constantine-Paton, 2010).

Several experiments suggest that BDNF-induced synaptic modulation is selective for both active synapses and specific synapses. For instance, in hippocampal slices, exogenous application of BDNF enhanced synaptic responses (Figurov et al., 1996). Moreover, addition of BDNF to cultured hippocampal neurons potentiated glutamatergic but not GABAergic synapses from the same pre-synaptic neuron, suggesting that different terminals could be independently modified (Schinder et al., 2000). Although we should always be cautious in correlating *in-vitro* observations with physiological conditions found in neuronal circuits of an intact organism, these observations point to a potential mechanism allowing BDNF to promote different synaptic responses.

It is still unclear how a freely diffusible protein, such as BDNF, is able to attain local effects at synapses and to modulate specific synapses. However, it is known that glutamatergic and GABAergic synapses are different in terms of molecular composition, morphology and ultimately function (O'Rourke et al., 2012), and therefore may differentially regulate compartmentalization of signaling complexes. Various mechanisms have been proposed to explain BDNF-induced specific synaptic effects, including local synthesis and secretion of BDNF at active synapses, activity-dependent synthesis and transport of TrkB mRNA, and TrkB receptor insertion into the plasma membrane (Lu, 2003). On the other hand, BDNF secreted at active synapses may induce TrkB receptors relocation from extra-synaptic sites to particular membrane microdomains enriched in synaptic zones (Nagappan and Lu, 2005), which could represent a complementary mechanism to the ones mentioned above.

Indeed, Suzuki et al. (2004) were the first to demonstrate that BDNF stimulation of cultured neurons induced translocation of TrkB receptors in lipid rafts of detergent insoluble neuronal membranes. Most importantly, this translocation was dependent upon TrkB autophosphorylation and it was accompanied by increased phosphorylation of ERK in lipid rafts, thus suggesting specific activation of the MAPK pathway (Suzuki et al., 2004). In addition, they found that BDNF-induced translocation and activation of TrkB in lipid rafts was critically relevant for neurotransmitter release and synaptic plasticity, since raft disruption by cholesterol depletion abolished the acute potentiating effect of BDNF on evoked synaptic transmission in culture, and the enhancement of the synaptic response to tetanus in hippocampal slices (Suzuki et al., 2004). However, TrkB translocation to lipid rafts was not important for neuronal survival. Treatment of neuronal cortical cultures with cholesterol synthesis inhibitors, known to effectively deplete lipid rafts, induced cell death, whereas addition of BDNF significantly enhanced cell viability in these conditions. This suggests that lipid rafts are not required for BDNF/TrkB signaling effect on neuronal survival. Thus, TrkB receptors in lipid rafts initiate local signaling, whereas, outside lipid rafts, they may activate alternative pathways, possibly involved in retrograde signaling (Suzuki et al., 2004). Regarding these, there are numerous studies (extensively reviewed elsewhere Harrington and Ginty, 2013), highlighting the importance of retrograde signaling of neurotrophin/Trk receptors to control the survival of different neuronal populations such us sympathetic and sensory neurons. In addition, Trk effectors, for example PI3K–AKT and Erk5, have been suggested to promote survival of neurons supported by target-derived neurotrophins (Harrington and Ginty, 2013). Therefore, we can speculate that depending on TrkB localization and local availability of effector molecules similar signaling pathways could alternatively be activated outside of lipid rafts.

Several components of pre- and post-synaptic regions have been described in lipid rafts, including ionotropic receptors as well as G-protein coupled receptors and their effectors (reviewed in Allen et al., 2007). Lipid rafts are also believed to contribute to trafficking of these receptors to and from the cell membrane (Pediconi et al., 2004), to their stability at the cell surface, and to ligand binding efficacy (Allen et al., 2007). In addition, similarly to BDBF/TrkB, also neurotransmitter receptors have been suggested to perform different functions depending on their localization in lipid rafts or outside these lipid microdomains. For example, NMDA receptors have been shown to mediate neurotoxicity when recruited within lipid rafts (Frank et al., 2004), whereas outside these microdomains they stimulate growth cone guidance (Guirland et al., 2004).

Although lipid rafts are enriched at synapses (Nagappan and Lu, 2005), whether BDNF/TrkB signaling role in lipid rafts occurs at synapses and whether it takes place pre-synaptically and/or post-synaptically remains to be elucidated. Not only BDNF is secreted from pre-synaptic terminals and post-synaptic boutons, but also TrkB receptors are present on the membrane on either side of the synaptic cleft (Edelmann et al., 2013).

In terms of signal transduction mechanisms, it was suggested that BDNF facilitates neurotransmitter release via the TrkB/MAPK/synapsin I signaling pathway (Jovanovic et al., 2000). Moreover, BDNF was found to stimulate cholesterol biosynthesis and increase the level of pre-synaptic proteins in lipid rafts (Suzuki et al., 2007). Taken together, these observations would support a role of BDNF/TrkB signaling at pre-synaptic terminals.

To our knowledge, so far no studies have specifically addressed BDNF-induced activation of TrkB receptors in the context of their partitioning at post-synaptic rafts, although a plethora of studies attributes a role of post-synaptic BDNF-TrkB signaling in synapse formation and plasticity (Yoshii and Constantine-Paton, 2010).

It has been shown that BDNF-TrkB signaling is required to deliver PSD95 to the post-synaptic membrane via vesicular transport (Yoshii and Constantine-Paton, 2007) and for the palmitoylation of PSD95 via activation of PLCγ and the brain-specific PKC variant protein kinase ζ (Yoshii et al., 2011). Interestingly, palmitoylation of proteins is a post-translational modification which is known to facilitate sequestration of proteins to lipid rafts (Linder and Deschenes, 2007). For instance, palmitoylation of the A-kinase anchoring protein 79/150 (AKAP79/150 is important to target AMPA receptors to post-synaptic rafts together with PSD-95, inducing synaptic potentiation (Keith et al., 2012).

**Figure 1:**
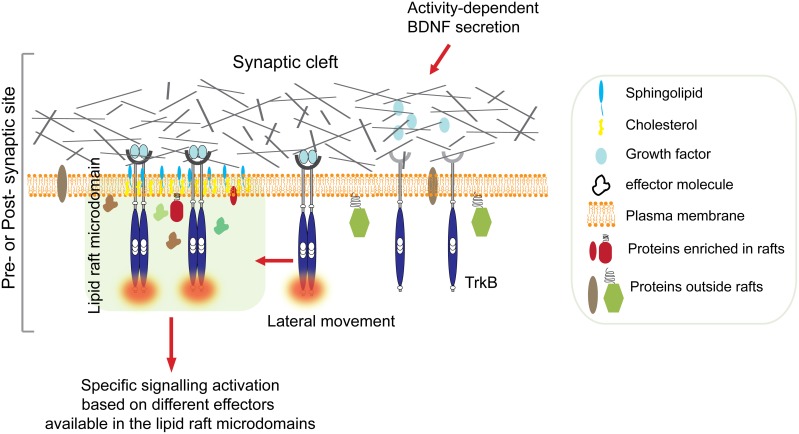
**Ligand-induced TrkB translocation into lipid raft at active synapses**. Synaptic activity stimulates BDNF secretion from both pre-synaptic terminals and post-synaptic boutons. High concentration of BDNF in the synaptic cleft is followed by TrkB receptors autophosphorylation and translocation into lipid rafts on membranes of either sides of the synaptic cleft. This is followed by activation of specific signaling depending on the kind of effectors available in the raft microdomain. For example, translocation of activated TrkB in lipid rafts at the pre-synaptic site stimulates the MAPK/synapsin I signaling pathway promoting neurotransmitter release (Jovanovic et al., 2000). In contrast, translocation of TrkB receptors to the post-synaptic site promotes palmitoylation and delivery of PSD95, and possibly other key molecules, to post-synaptic membrane rafts via activation of PLC-γ and PKC pathways thus promoting synaptic plasticity (Yoshii et al., 2011).

Therefore, one can hypothesize that BDNF-stimulated TrkB signaling could potentially coordinate the delivery of key molecules at post-synaptic lipid rafts to promote synaptic plasticity (Figure 1). This idea is supported by combined *in vitro* and *in vivo* observations that provide the first genetic evidence for a role of the non-receptor tyrosine kinase Fyn in the selective BNDF-induced translocation of TrkB receptors to intracellular lipid rafts (Pereira and Chao, 2007).

Internalization of the BDNF/TrkB receptor complex is an important step in the regulation of TrkB signaling, and of neurotrophin retrograde signaling in general (Harrington and Ginty, 2013). Fyn is a member of the Src family of tyrosine kinases protein, which has been previously shown to co-immunoprecipitate with TrkB receptors and to localize in lipid rafts (Iwasaki et al., 1998).

In Fyn knockout neurons, after BDNF stimulation, TrkB receptors translocation in lipid rafts was reduced and this was accompanied by diminished activation of the PLC-γ pathway (Pereira and Chao, 2007). Moreover, Fyn knockout mice have severe neurological defects, including deficits in hippocampal LTP (Grant et al., 1992), which are similar to those found in mouse mutants where the BDNF/TrkB/PLC-γ pathway is genetically impaired (Patterson et al., 1996; Minichiello et al., 2002).

Taken together, these observations illustrate an important mechanism mediated by Fyn in facilitating BDNF/TrkB induced PLC-γ signaling cascade, possibly at synapses, through receptor localization in intracellular lipid rafts. However, these findings are in contrast to Suzuki and colleague's observations, in which BDNF-induced TrkB receptor activation in lipid rafts stimulated the ERK/MAPK pathway (Suzuki et al., 2004). The discrepancy is likely to be due to differences in the sensitivity of their detection methods, in the employment of pure versus mixed neuronal populations, and/or in the amount of exogenous BDNF stimulation applied to their respective neuronal cultures. Possibly the relative abundance of BDNF may account for the selective activation of signaling pathways depending on TrkB receptor enrichment in lipid rafts either at the cell surface or in internal compartments. Indeed, using antibody cross-linking, TrkB receptor colocalizing with lipid rafts was also visualized on the surface of living cells (Pereira and Chao, 2007).

## NGF-induced signaling at synaptic rafts

There is no direct evidence for a role of NGF signaling at synaptic rafts. Because of its low abundance in the CNS, NGF action at synapses has been studied prevalently in sensory neurons. However, evidence for activity dependent-release of NGF in the hippocampal-septal system has been recently provided (Guo et al., 2012). Both TrkA and p75^NTR^ receptors were found enriched in lipid raft membranes (Peiro et al., 2000; Higuchi et al., 2003; Pryor et al., 2012). In neural crest-derived PC12 cells, NGF treatment stimulates translocation of TrkA receptors to lipid rafts and this localization is required for activation of ERK. Translocation appears to be mediated by association with flotilin, a component of lipid rafts, and the adapter protein c-CbI associated protein (CAP) (Limpert et al., 2007). Although the biological activity of TrkA receptors in PC12 cells are likely to differ from those found in neurons, these observations underscore a potential common mechanism among neurotrophins in organizing and compartmentalizing signal transduction.

Recent evidence points to a possible role of NGF signaling in organizing the association of lipid rafts with the cytoskeleton (Pryor et al., 2012). Indeed, the latter plays an essential role in stabilizing specific sets of proteins localized at the plasma membrane, in molecular trafficking, cell adhesion, migration and remodeling of growth cones and synapses (Tolias et al., 2011). Cytoskeletal proteins also interact with specific lipids (Janmey and Lindberg, 2004) and are enriched in lipid rafts. For example, RhoGTPases have been isolated from insoluble membrane fractions (Dupree and Pomicter, 2010) and are considered key regulators of the actin cytoskeleton, playing an important role in synapse remodeling (Tolias et al., 2011).

Pryor et al. (2012) have recently shown *in vitro* that NGF specifically enhances the binding of TrkA, but not p75^NTR^, to microtubules in detergent resistance membranes, and that this binding does not require phosphorylation of TrkA (Pryor et al., 2012). Since activation of neurotrophin receptors in endosomes is required for their trafficking (Harrington and Ginty, 2013), this finding suggests a possible mechanism for local signaling that is distinct from the one associated with retrograde transport. Indeed, in PC12 cells Rac1, a member of small GTPases, was targeted to lipid rafts when activated by NGF. In addition, Rac1 activation was impaired by treatment with a cholesterol-sequestering agent (Fujitani et al., 2005). These observations indicate that reorganization of the cytoskeleton at specific sites in the membrane might be controlled by NGF-dependent targeting of key molecules to lipid rafts. However, further studies are needed to elucidate this mechanism and the specific neurotrophin receptors involved (Harrington and Ginty, 2013).

## Concluding remarks

In summary, the literature reviewed here is alluding to the idea that neurotrophin signaling may occur by recruitment of neurotrophin receptors at specific sites on the cell surface and/or in internal compartments that might be distinct from signaling endosomes. This recruitment, if occurring at synaptic rafts, might elicit activation of distinct signaling pathways involved in synaptic plasticity.

In the context of neurotrophin signaling, the lipid raft hypothesis is quite attractive. One can speculate that the temporally controlled availability of neurotrophins at synapses can stimulate lipid rafts to function as traffic lights, directing the spatial recruitment and time of activation of specific receptors and effector molecules with the exclusion of others. In doing so, they could potentially fulfil a critical role in modulating and fine-tuning synaptic signaling, by partitioning signal transduction mechanisms along the synaptic and extra-synaptic membranes, and within the synapse itself. In addition, since lipid rafts are highly dynamic they can bring together various combinations of adaptor molecules and enzymes whose availability could depend on the strength of synaptic activity and the cell type, adding an additional level of specificity in time and space (Simons and Toomre, 2000).

However, the studies presented here did not use synaptosomal preparations and were based on the assumption that the current methods used to isolate raft–associated proteins and to study their biological functions are valid methods. Nevertheless, these methods are prone to produce conflicting or inconsistent results because they cannot capture the level of complexity predicted by the idea that lipid rafts are fluctuating nanoscale entities and therefore may vary in composition in a timescale that cannot be resolved by available tools. Therefore, progress in this active area of research will depend on the development of new technologies to study lipid rafts biology especially *in vivo*.

Whether lipid rafts coordinate neurotrophin signaling at synapses *in vivo* is assumed but not demonstrated. Only technological advancement will test this idea, and as Karl Popper's says: “Every genuine test of a theory is an attempt to falsify it, or to refute it. Testability is falsifiability; but there are degrees of testability: some theories are more testable, more exposed to refutation, than others; they take, as it were, greater risks” (Popper, 1963).

### Conflict of interest statement

The authors declare that the research was conducted in the absence of any commercial or financial relationships that could be construed as a potential conflict of interest.
